# Comparison of standard coagulation testing with thromboelastometry tests in cardiac surgery

**DOI:** 10.15171/jcvtr.2019.48

**Published:** 2019-10-13

**Authors:** Elham Khalaf- Adeli, Mostafa Alavi, Alireza Alizadeh-Ghavidel, Ali Akbar Pourfathollah

**Affiliations:** ^1^Blood Transfusion Research Center, High Institute for Research and Education in Transfusion Medicine, Tehran, Iran; ^2^Rajaie Cardiovascular Medical and Research Center, Iran University of Medical Sciences, Tehran, Iran; ^3^Heart Valve Disease Research Center, Rajaie Cardiovascular Medical and Research Center, Iran University of Medical Sciences, Tehran, Iran; ^4^Departments of Immunology, Faculty of Medicine, Tarbiat Modares University of Medical Sciences, Tehran, Iran

**Keywords:** Cardiovascular surgery, Thromboelastometry, Blood transfusion

## Abstract

***Introduction:*** According to the several evidences, using thromboelastometry as a point of care test can be effective in reduction in blood loss and transfusion requirements in cardiac surgeries. However, there are limited data regarding to the comparison of thromboelastometry and the standard coagulation tests. In this study, we compared thromboelastometry and standard coagulation tests (PT, PTT and fibrinogen level) in patients under combined coronary-valve surgery.

***Methods:*** Forty adult patients who were under on-pump combined coronary-valve surgery were included in this study. Thromboelastometry tests Fibtem, Intem, Extem and Heptem), along with standard coagulation tests (PT, PTT and fibrinogen assay) were simultaneously performed in two time points, before and after the pump (pre-CPB and post-CPB, respectively).

***Results:***A total of 80 blood samples were analyzed. There were no significant correlation between PT test and the CT-Extem parameter as well as PTT and CT-Intem parameter either in pre-CPB and post-CPB (*P* >0.05). On the contrary, fibrinogen level had high correlation with A10-Fibtem and A10-Extem in pre-PCB (*P* <0.05). 82% of PT and 84% of PTT measurements were outside the reference range, while abnormal CT in Extem and Intem was observed in 17.9%.

***Conclusion:*** For management of bleeding, adequate perioperative haemostatic monitoring is indispensable during cardiac surgery. Standard coagulation tests are time consuming and cannot be interchangeably used with thromboelastomety and relying on their results to decide whether blood transfusion is necessary, leads to the inappropriate transfusion.

## Introduction

Cardiac surgeries with cardiopulmonary bypass (CPB) expose the patients to an increased risk of heavy bleeding, necessitating the need for blood transfusion, as a result of the acquired coagulopathy. Several studies have been reported that the contact of blood with CPB circuit, leads to the over-activation of the coagulation system, thereby, overusing the coagulation factors during the operation. On the other hand, administration of heparin in large amounts as an anticoagulant inhibits the function of some coagulation factors.^1-4^ Patients undergoing combined coronary-valve surgery are more susceptible to heavy bleeding due to the prolonged period of the operation and CPB. Standard coagulation tests such as PT, PTT, and in some cases, assay of fibrinogen concentration, are available for the assessment of coagulation system. Each test assesses the function of a part of the coagulation system and requires a long processing time. This, in turn, hinders the timely management of coagulopathy in patients.^5^ The complicated nature of coagulopathy in one hand, and the delay in access to the results of traditional coagulation tests on the other, result in empirical decision making, rather than practical, for the correction of coagulopathy.

In recent studies, different types of Point-of-care coagulation testing methods like thromboelastometry, were highly considered for the diagnosis of coagulation in patients under different types of surgeries. As a matter of convenience, thromboelastometry tests may be brought to the patient’s bedside. These tests provide the possibility of evaluation of the clot formation kinetic from intrinsic and extrinsic pathway. Furthermore, these tests can be carried out with whole blood samples, which eliminates the need for processing the samples, giving the physician access to the results more quickly. With regard to the dynamic status of the coagulation system in patients undergoing surgery, thromboelastometry tests also help physicians make proper medical decisions for the patient. Studies have suggested that thromboelastometry is effective in alleviating the hemorrhage and facilitating the administration of blood products in cardiac surgeries.^6-10^ However, there are limited data regarding to the comparison of thromboelastometry and the standard coagulation tests.

In the present study, we compared thromboelastometry and standard coagulation tests (PT, PTT and fibrinogen level) in patients under combined coronary-valve surgery.

## Materials and Methods

The Statistical sample included 40 adult patients who were under on-pump combined coronary-valve surgery in Shahid Rajaie Cardiovascular, Medical and Research Center. Thromboelastometry tests (Fibtem, Intem, Extem and Heptem), along with standard coagulation tests, such as PT, PTT and fibrinogen assay, were performed in two time points, before and after the application of pump (pre-CPB and post-CPB, respectively). Post-CPB tests were performed 15 minutes after the reversal of heparin with protamine sulfate. Briefly, 2.7 mL of venous blood was collected in tubes containing 0.3 mL of the anticoagulant sodium citrate 3.2% (Becton Dickinson, USA) to perform thromboelastometry by means of a rotational thromboelastometry (ROTEM, TEM International GmbH, Munich, Germany) device before and after pumping. Blood samples required for the standard coagulation tests were collected in tubes containing sodium citrate 3.2%, at the same time. Quantitative assay of fibrinogen was performed with Clause’s method. All the standard coagulation tests were conducted using a coagulometer (Stago, France). The results included values outside of the reference range were considered abnormal. In comparative investigations on the standard coagulation tests and thromboelastometry, CT-Extem, CT-Intem, and A10-Extem/A10-Fibtem parameters were compared to PT, PTT and Fibrinogen, respectively. The collected data were analyzed with IBM SPSS 22 (IBM Inc., Armonk, NY). The values of qualitative and quantitative data were represented as (absolute count) percentage and mean ± SD. Paired-sample *t* test was used to estimate the changes in pre and post CPB time points. Presence of any correlation between the variables was investigated using Pearson correlation coefficient. All the results were considered significant, with *P* values below 0.05.

## Results

### The results of coagulation tests in pre-CPB and post-CPB time points

Patients characteristics in the beginning of the study and intraoperative variables are represented in Table 1. Platelet count in all the 40 patients was measured to be 100 000/mm^3^ and above on arrival at the operation room. A total of 80 ROTEM tests were conducted to monitor the coagulation system in patients under study. Table 2 shows the mean values of the results from standard coagulation tests and thromboelastometry for each parameter. It can be inferred from the table that the mean value for CT parameter in Extem, Intem and Heptem tests increased significantly in post-CPB time point compared to the values reported for the pre-CPB time point. The post-CPB mean value for A10 parameter in both Extem and Fibtem tests decreased significantly, in comparison to pre-CPB time point. The mean value for PT and PTT increased remarkably in post-CPB time point, compared to the values recorded before the application of CPB. Unlike the latter, the mean value for fibrinogen level showed a significant decline in post-CPB time point, compared to the level recorded in pre-CPB time point. The change reported for results of all coagulation tests, including both standard and thromboelastometry tests, was statistically significant (*P* < 0.05).

**Table 1:** Patient’s characteristics and preoperative laboratory tests results

**Variable**	**Value**
Female	26 (64%)
Male	14 (36%)
Age (y)	58±11
Weight (kg)	67±11
Hb (g/dL)	12.8±1.8
PT (s)	14.4±1.5
INR	1.14±0.15
PTT (s)	34±16
Platelet count (x 10^9^/L)	242±75
CPB time (min)	114±44
Aorta clamp time	60±5
ACT (after heparin revers) (s)	127±16
Anemic patients (%)	12 (31%)

Abbreviation: Hb; hemoglobin, PT; prothrombin time, PTT; partial thromboplastin time, CPB; cardiopulmonary bypass, ACT; activated clotting time. The results are expressed as absolute number (percentage) or mean ± SD.

**Table 2:** Comparison of the coagulation tests results in pre- and post-CPB time point

**Parameter**	**Pre-CPB**	**Post-CPB**	***P*** **value**
CT-Extem (s)	64 (11)	73 (10)	<0.001*
A10-Extem (mm)	61 (7)	49 (11)	<0.001*
CT- Intem (s)	175 (25)	214 (49)	<0.001*
A10-Fibtem (mm)	21 (6)	14 (5)	<0.001*
CT-Heptem (s)	175 (33)	190 (38)	0.001*
PT (s)	18.7 (4)	24.9 (14)	0.007*
PTT (s)	45 (14)	64 (24)	<0.001*
Fibrinogen (mg)	317 (82)	231 (91)	<0.001*
Di-Dimer	0.77 (0.57)	1.9 (1.6)	*<0.01

Abbreviation: PT; prothrombin time, PTT; partial thromboplastin time, CT; clotting time, A10; Amplitude 10. Significant results with *P* values below 0.05 are shown with star sign.

### Comparison ofthromboelastometryand standard coagulation tests

**Figure 1:**
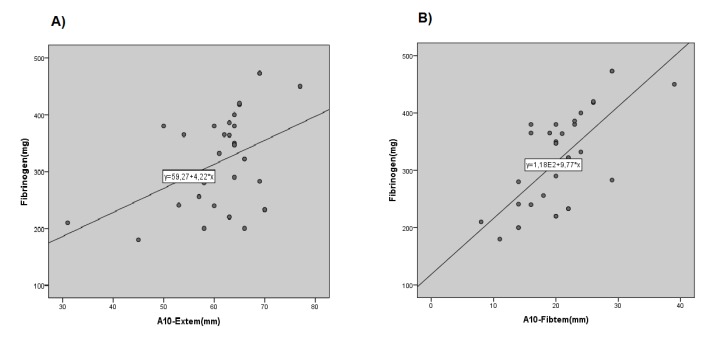
The correlation between fibrinogen levels and A10-Extem (A) / A10-Fibtem (B) in pre-CPB time point.

To investigate the relationship between the results from thromboelastometry and the standard coagulation tests, the results for PT, PTT and fibrinogen tests were compared to corresponding parameters from thromboelastometry. As CT parameter in Extem and Intem represents the data of clot formation through intrinsic and extrinsic pathways, respectively, the results from these tests were compared to those of PT and PTT. Also, since the parameters of A10-Extem and A10-Fibtem reflect the involvement of fibrinogen in clot formation, they were compared to fibrinogen level. The correlation between the results from thromboelastometry and standard coagulation tests was determined with Pearson’s correlation coefficient test. *P* and *R* values are listed in Table 3. Based on the results obtained, there was no significant correlation between PT test and the CT-Extem parameter either before or after CPB (*P* > 0.05). Similarly, PTT test and the CT-Intem parameter were not shown to have any significant relationship in any time points (*P* > 0.05). On the contrary, fibrinogen level had high correlation with A10-Fibtem and A10-Extem in pre-PCB, respectively (*P* < 0.05; Figure 1). The analysis of data suggested that 38% (n=15) of patients had abnormal pre-CPB PT results, which after pumping increased to 82% (n=32). However, based on the reference values for CT-Extem, the fraction of patients with abnormal coagulation test results at identical times was 2.5% (n=1) and 17.9% (n=7), respectively. Likewise, 41% (n=16) of patients had abnormal PTT results before pumping, that ultimately increased to 84% (n=33) after the pumping. Therefore, based on the reference values for CT-Intem, the prevalence of patients with abnormal results in pre and post-CPB time point was 0% and 17.9% (n=7), respectively.

**Table 3:** Correlation between thromboelastometry and the standard coagulation tests. Both *P* value and *R* value are presented

**Conventional coagulation test**	**Rotemvariable**	**Pre-CPB**		**Post-CPB**	
		***P*** **value**	***R*** **value**	***P*** **value**	*R* value
PT	CT- Extem	0.9	0.01	0.3	0.06
PTT	CT- Intem	0. 2	0.19	0.06	0.3
Fibrinogen	A10-Extem	0.01*	0.4	0.2	0.22
	A10-Fibtem	0.001*	0.69	0.1	0.26

Abbreviations: PT; prothrombin time, PTT; partial thromboplastin time, CT; clotting time, A10; amplitude 10. Significant results with *P* values below 0.05 are shown with star sign.

Furthermore, according to the reference values for fibrinogen level, the prevalence of patients with abnormal results before and after pumping, was 2.5% (n=1) and 35% (n=14), respectively. Finally, the prevalence of abnormal patients, based on the reference values for A10-Fibtem at identical times, was 0% and 15% (n=6), respectively.

## Discussion

Several studies have been conducted to investigate the role of thromboelastometry tests in management of coagulopathy in patients under cardiac surgery.^6-8^ The present study aimed to compare the results of routine coagulation tests with those of thromboelastometry in patients undergoing combined coronary-valve surgery.

Extem and Intem tests examines the process of clot formation through the extrinsic and intrinsic pathway of coagulation, respectively. CT parameter in both tests increased significantly in post-CPB time point. An increase in the value of CT parameter for both pathways indicates that CPB may have an effect on decline in the level of coagulation factors or their function. In addition, the comparisons suggested that the value of A10-Fibtem parameter decreased significantly in post-CPB time point. Since a platelet inhibitor is usually employed to perform the Fibtem test, this test is specifically used for the evaluation of the role of fibrinogen in clot formation. It should be noted that parallel to the significant changes in CT-Extem and CT-Intem parameters, the results from PT (investigating on the extrinsic pathway of coagulation) and PTT tests (dealing with the intrinsic pathway of coagulation) are indicative of a significant increase in post-CPB time point. Moreover, parallel to a remarkable decrease in A10-Fibtem parameter value after CPB, a drastic decline is also present in the fibrinogen level. These results are consistent with those of Espinosa et al’s study that investigated the changes resulting from CPB in different POC tests. However, in the latter, changes arising from CPB in CT-Extem results were not reported.^11^

Consistent with the results reported by Espinosa et al our findings confirmed the decline in the level of coagulation factors during CPB, which had been noted by previous studies.^2,12,13^ Although CPB induced considerable changes in the values of parameters mentioned earlier, the mean of the values measured in post-CPB time point remained in the normal range.

In a study that investigated the relationship between the changes in coagulation factors and post- operative hemorrhage, Gelb et al suggested that the decline in the level of coagulation factors during CPB or following the procedure, was not correlated with extensive bleeding.^2^ Therefore, it seems despite the fact that CPB has an influence on the coagulation system, these changes may not reach the abnormal level in every patient, and thus they may not be necessarily related to associated with post-operative hemorrhage. Accordingly, in most cases there is no need for prophylactic interventions with blood products.

Based on the results obtained in this study, the values for CT-Extem and CT-Intem in the post-CPB time point, increased by 14% and 22%, respectively, in relation to pre-CPB values. Also, in the present study, the value of A10-Fibtem parameter decreased by 33%, showing the highest rate of change among all the parameters measured. In a non-systematic study that explored the changes in different coagulation test results in various studies on cardiac surgeries, Hofer et al reported that the increase in the value of CT-Extem parameter fell in the range of 11.5% to 48%. Such a broad spectrum of changes may result stem from differences in the types of surgeries and intraoperative approaches. However, Hofer et al, as well as other researchers, did not reported changes in CT-Extem and A10-Fibtem parameters.^12^

The correlation between the results from thromboelastometry and traditional coagulation tests was determined with Pearson’s correlation coefficient. According to our data, there was not any significant correlation between PT and PTT test results with CT-Extem and CT-Intem parameters in neither time points (pre and post-CPB), which is consistent with results from the study by Espinosa et al. In their study, Espinosa and colleagues reported a high correlation between fibrinogen level and Fibtem values. In the present study, there was also a significant high correlation between A10-Fibtem values and fibrinogen level in pre-CPB time point. It should be noted that Espinosa et al’s study involved 35 patients undergoing elective cardiac surgery, of whom three patients had coronary-valve surgery. Moreover, in their study, the patient was under CPB for less than 100 minutes on average. In the present study, the average time of CPB procedure was estimated to be over 100 minutes.^11^

While abnormal values of CT-Extem were observed in 17.9% of the patients in post-CPB time point, 82% of the patients showed increased PT (above the normal range) at the same time. In addition, 84% of patients had increased PTT, whereas 18% of patients showed elevated values for CT-Intem. Moreover, the prevalence of patients with abnormal results, based on the reference values of A10-Fibtem, was 15% in post-CPB time point, while 35% of patients had less than normal fibrinogen, based on the reference values for fibrinogen level. Such disparity might have resulted from the fact that the traditional coagulation tests are performed on plasma, whereas thromboelastometry tests requires complete blood samples. PT and PTT tests are traditionally used for diagnosing coagulation disorders and hemostatic therapy with blood products, especially, plasma.

It should be noted that despite the different reports around the clinical inclination to administer of FFP in large amounts, during the past two decades, there is actually little evidence indicating the usefulness of the application of this product in treatment of bleeding.^14-16^ On the other hand, different studies have suggested that PT and PTT tests do not have high predictive value to determine the clinically important forms of bleeding.^17^ Cammerer et al and Davidson et al suggested that thromboelastometry tests had a high negative predictive value in predicting the likelihood of serious postoperative bleeding in patients.^18,19^

Traditional tests investigate the coagulation system under the static conditions, offering only the data regarding the primary formation of thrombin and fibrin, while a large portion of thrombin and coagulation factors are activated as the coagulation cascades progress. For this reason, such tests cannot represent a close-to-reality image from the status of coagulation system under surgical conditions. On the other hand, the physician usually have to wait for a long period time for the laboratory results, since traditional tests performed using plasma, which needs to be processed before the tests are conducted. The long turnaround time in many cases, make the physician take an empirical decision for blood transfusion. According to the results reported by Toulon et al, the average turnaround time for traditional coagulation test results is about 40-60 minutes.^5^ In the present study, the results of thromboelastometry tests were prepared and reported in 15-20 minutes.

With regard to the entire results from this study and the others, it seems that relying on traditional coagulation test results to decide whether blood transfusion is necessary, leads to the inappropriate transfusion, ultimately increasing the volume of transfusion, especially regarding the plasma products. Therefore, application of thromboelastometry tests for hemostatic management can be useful inacceleration of critical judgment in clinical decision making, as well as avoiding the transfusion of unnecessary blood products, offering a more satisfactory care for the patients.

## Competing interests

None to be declared.

## Ethical approval

This study was approved by the both ethics committee of Iranian blood transfusion organization (ethical code IR.TMI.REC.1393.14) and Rajaie cardiovascular medical and research center (ethical code: RHC.AC.IR.REC.1394.29).
